# Fructus Gardenia Extract Ameliorates Oxonate-Induced Hyperuricemia with Renal Dysfunction in Mice by Regulating Organic Ion Transporters and mOIT3

**DOI:** 10.3390/molecules18088976

**Published:** 2013-07-29

**Authors:** Qing-Hua Hu, Ji-Xiao Zhu, Jing Ji, Lin-Lin Wei, Ming-Xing Miao, Hui Ji

**Affiliations:** 1 State Key Laboratory of Natural Medicines, China Pharmaceutical University, Nanjing 210009, China; 2 Chinese Medicine Germplasm Resource Engineering Technology Research Center of Jiangxi Province, Jiangxi University of Traditional Chinese Medicine, Nanchang 330004, China

**Keywords:** Fructus Gardenia Extract, hyperuricemia, renal dysfunction, renal organic ion transporters, mOIT3

## Abstract

The potent anti-hyperuricemia activities of Fructus Gardenia Extract (FGE) have been well reported. The aim of this study was to evaluate the uricosuric and nephro-protective effects of FGE and explore its possible mechanisms of action in oxonate-induced hyperuricemic mice. FGE was orally administered to hyperuricemic and normal mice for 1 week. Serum and urinary levels of uric acid, creatinine and blood urea nitrogen (BUN), and fractional excretion of uric acid (FEUA) were measured. The mRNA and protein levels of mouse urate transporter 1 (mURAT1), glucose transporter 9 (mGLUT9), ATP-binding cassette, subfamily G, 2 (mABCG2), organic anion transporter 1 (mOAT1), mOAT3, oncoprotein induced transcript 3 (mOIT3), organic cation/carnitine transporters in the kidney were analyzed. Simultaneously, Tamm-Horsfall glycoprotein (THP) levels in urine and kidney were detected. FGE significantly reduced serum urate levels and increased urinary urate levels and FEUA in hyperuricemic mice. It could also effectively reverse oxonate-induced alterations in renal mURAT1, mGLUT9, mOAT1 and mOIT3 expressions, as well as THP levels, resulting in the enhancement of renal uric acid excretion. Moreover, FGE decreased serum creatinine and BUN levels, and up-regulated expression of organic cation/carnitine transporters, improving renal dysfunction in this model. Furthermore, FGE decreased renal mABCG2 expressions in hyperuricemic mice, contributing to its beneficial actions. However, further investigation is needed in clinical trials of FGE and its bioactive components.

## 1. Introduction

Hyperuricemia, a key risk factor for the development of gout, has been involved in many diseases such as renal dysfunction, cardiovascular diseases, hypertension, hyperlipidemia, diabetes and metabolic syndrome [1,2]. Impaired renal excretion of uric acid rather than uric acid overproduction, however, was considered to be the major cause of hyperuricemia [3]. Urate transport-related proteins, such as organic anion transporters involved in renal urate secretion and re-absorption, play a pivotal role in maintaining urate homeostasis *in vivo* [4,5].

Renal tubular handling of uric acid is dependent on tubular transporters [6]. Some organic anion transpoters, such as urate transporter 1 (URAT1, SLC22A12) located at the apical membranes, have been reported to be responsible for proximal tubule transport of uric acid, accounting for urate re-absorption [7]. Glucose transporter 9 (GLUT9, SLC2A9) has two splice variants, both of which have been proposed to mediate urate re-absorption [8]. The two splice variants differ only in the length of their amino-terminal cytoplasmic domains, locating at the basolateral (hGLUT9) and apical sides (hGLUT9△N) of proximal convoluted tubules, respectively [9,10]. Two splice variants of mouse GLUT9 (mGLUT9a and mGLUT9b) share the same transport characteristics of hGLUT9, but mainly distributed in distal connecting tubules [11]. ATP-binding cassette, subfamily G, 2 (ABCG2) present at the apical membrane, has been proved to be involved in renal secretory transport of uric acid [12]. Moreover, organic anion transporter 1 (OAT1, SLC22A6) and OAT3 (SLC22A8) distributed at the basolateral membrane contribute to renal urate secretion by mediating urate transport across cell membranes in kidney proximal tubules [13]. Given altered expressions of these organic anion transporters observed in hyperuricemic rodents, the recovery of urate transpoter expressions may exert positive effects on hyperuricemia improvement. Recently, the oncoprotein induced transcript 3 (OIT3), also named liver-specific zona pellucida domain-containing protein (LZP), has been reported to maintain urate homeostasis by regulating the excretion and re-absorption of uric acid in renal tubule via cooperating with Tamm-Horsfall glycoprotein (THP) [14]. Nevertheless, there were few studies focusing on the role of OIT3 in renal urate excretion in hyperuricemic individuals. On the other hand, hyperuricemia is known to cause renal dysfunction, which can be indicated by renal tubular transport of organic cations mediated by renal organic cation and carnitine transporters (OCTs and OCTNs) [15,16]. The reduced expression of renal OCTs and OCTNs is observed in rats or mice with various renal injuries [17,18,19], therefore indicating that regulation of renal OCTs and OCTNs expressions might exert nephroprotective effects on hyperuricemia with kidney dysfunction.

Clinically, hypouricemic agents including uricosuric agents and xanthine oxidase inhibitors are available. Unfortunately, both of them could produce unacceptable adverse effects, such as allpurinol-induced hypersensitivity syndrome [20] and benzbromarone-induced hepatotoxicity [21]. Thus, it is necessary to explore safer and more effective hypouricemic agents. Fructus Gardenia, the dried ripe fruit of *Gardenia jasminoides* (Ellis.), is used clinically against inflammation, viral encephalitis, hepatitis, tonsillitis, tracheitis and high fever, as recorded in the State Pharmacopoeia of People’s Republic of China (2010 edition). Our previous study suggested that FGE could attenuat oxonate-induced hyperuricemia in mice through regulating hepatic xanthine oxidase, nevertheless, the potential of FGE in down-regulating xanthine oxidase activities were not parallel with that in lowering serum uric acid levels [22]. Thus, further investigation is needed in clarifying the exact mechanisms of FGE in improving hyperuricemia. Here, we assessed the hypouricemic effects of FGE with respect to its possible mechanisms by determining the expression levels of renal urate transport-related genes, such as mURAT1, mGLUT9, mABCG2, mOAT1, mOAT3 and mOIT3. In order to evaluate the effects of FGE on renal function in the setting of hyperuricemia, we determined serum creatinine and blood urea nitrogen (BUN) levels, and creatinine clearance (Ccr). Simultaneously, the expression levels of renal organic cation and carnitine transporters, as well as THP levels were examined in FGE-treated hyperuricemic and normal mice.

## 2. Results

### 2.1. Effects of FGE on Serum Uric Acid Levels, Renal Excretion of Uric Acid and Renal Function

As shown in Table 1, after seven consecutive days of 250 mg/kg/day oxnate administration, serum uric acid levels of mice were obviously elevated compared with the normal control group. Serum uric acid levels in hyperuricemic animals were significantly lowered by the treatment of FGE at 139, 278 and 556 mg/kg in a dose-dependent manner. In addition, positive control group allopurinol significantly decreased the levels in this model, even lower than that of normal group. On the other hand, potassium oxonate treatment remarkably reduced 24 h-urinary uric acid and creatinine excretion, and increased serum creatinine levels in mice. Notably, treatment of 278 and 556 mg/kg FGE and 5 mg/kg allopurinol recovered uric acid and creatinine excretion in 24 h, as well as serum creatinine levels in hyperuricemic mice. Moreover, FGE at 139 mg/kg also increased the decreased 24 h-urinary uric acid excretion in potassium oxonate-treated mice significantly. Fractional excretion of uric acid (FEUA), as a renal uric acid handling parameter, was remarkably decreased in potassium oxonate-induced hyperuricemic group in comparison with normal group, which could be restored by treatment of FGE and allopurinol at all doses. In addition, neither FGE nor allopurinol affected these biochemical parameters in normal mice, except that allopurinol at 5 mg/kg even significantly lowered uric acid levels in normal group, exhibiting potential side effects.

### 2.2. Effects of FGE on Renal mRNA and Protein Levels of Urate Transporters

**Figure 1:**
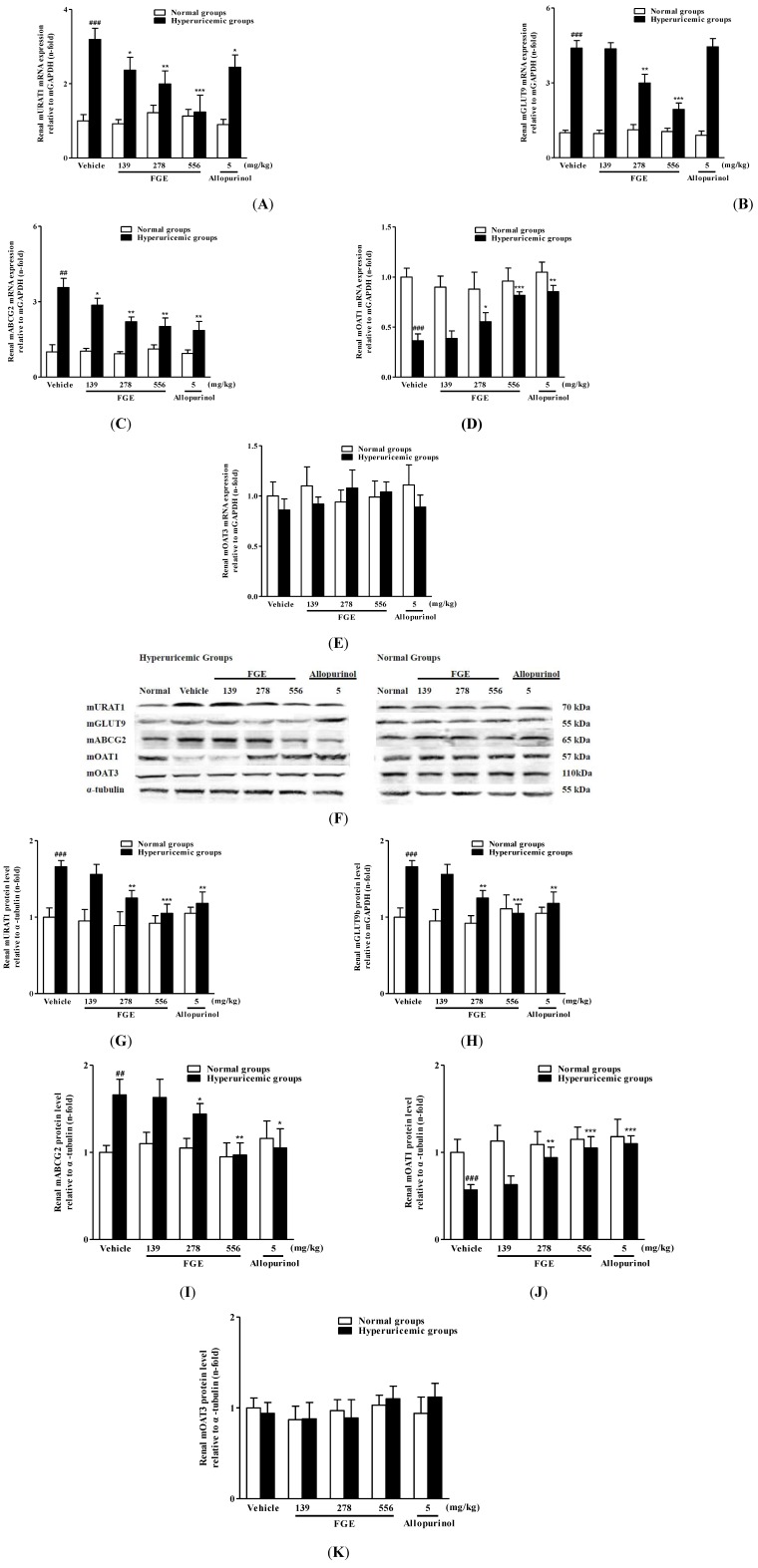
Effects of FGE and allopurinol on mRNA and protein levels of renal urate transport-related transporters in hyperuricemic and normal mice. Kidney cortex mRNA was extracted for RT-PCR analysis of mURAT1 (**A**), mGLUT9 (**B**), mABCG2 (**C**), mOAT1 (**D**) and mOAT3 (**E**). Kidney cortex tissues were used for western blot analysis of mURAT1, mABCG2, mGLUT9, mOAT1 and mOAT3 (**F**). The ratio of mURAT1 (**G**), mABCG2 (**H**), mGLUT9 (**I**) mOAT1 (**J**) and mOAT3 (**K**) density to α-tubulin density. Data were expressed as the mean ± S.E.M. for 4 mice. ^##^ *p* < 0.01, ^###^ *p* < 0.001, compared with normal vehicle animals. ^*^ *p* < 0.05, ^**^ *p* < 0.01, ^***^ *p* < 0.001, compared with hyperuricemia vehicle animals.

The effects of FGE and allopurinol on mRNA and protein levels of renal mURAT1, mGLUT9, mABCG2 and mOAT1 in hyperuricemic mice were shown in Figure 1. Potassium oxonate significantly up-regulated expressions of mURAT1, mABCG2, mGLUT9 and down-regulated expression of mOAT1, but failed to alter expression of mOAT3 in mouse kidneys. In potassium oxonate-treated mice, FGE at the two higher doses significantly restored the mRNA and protein expression levels of these renal urate transporters, while it only succeeded in down-regulting renal mRNA levels of mURAT1 and mABCG2 at 139 mg/kg. Allopurinol at 5 mg/kg succeeded in recovering both mRNA and protein expressions of renal mURAT1, mABCG2 and mOAT1, but failed to down-regulate renal mGLUT9 expressions in hyperuricemic mice. In addition, neither FGE nor allopurinol affected mRNA and protein expressions of renal mURAT1, mABCG2, mGLUT9, mOAT1 and mOAT3 in normal mice.

**Table 1:** Effects of FGE and allopurinol (AP) on serum uric acid levels (SUA), serum creatinine levels (SCr), excretion of uric acid (UUA) in 24 h, excretion of creatinine (UCr) in 24 h, fractional excretion of uric acid (FEUA) and blood urea nitrogen (BUN) concentrations in hyperuricemic and normal mice.

	Dose (mg/kg)	SUA (mg/dL)	SCr (mg/dL)	UUA (mg)	UCr (mg)	FEUA	BUN (mg/dL)
**Normal**							
Vehicle	-	3.88 ± 0.14	0.92 ± 0.08	0.398 ± 0.017	0.55 ± 0.05	19.0 ± 3.3	13.3 ± 0.3
FGE	139	3.96 ± 0.18	0.88 ± 0.05	0.373 ± 0.028	0.30 ± 0.04	18.4 ± 2.4	13.1 ± 0.2
	278	4.10 ± 0.15	0.95 ± 0.11	0.390 ± 0.016	0.38 ± 0.05	19.4 ± 2.5	14.0 ± 1.1
	556	3.77 ± 0.20	0.91 ± 0.07	0.351 ± 0.031	0.51 ± 0.07	18.2 ± 2.7	13.5 ± 0.4
	5	3.02 ± 0.12 ^***^	0.95 ± 0.07	0.389 ± 0.024	0.52 ± 0.01	19.5 ± 3.1	12.9 ± 0.6
**Hyperuricemia**							
Vehicle		5.31 ± 0.21 ^###^	1.25 ± 0.06 ^###^	0.215 ± 0.045 ^###^	0.28 ± 0.02 ^###^	14.3 ± 5.6 ^##^	16.5 ± 0.4 ^###^
FGE	139	4.36 ± 0.14 ^**^	1.08 ± 0.06 ^*^	0.243 ± 0.008	0.30 ± 0.04	16.4 ± 2.8 ^*^	15.3 ± 0.2 ^*^
	278	4.20 ± 0.12 ^***^	1.05 ± 0.09 ^**^	0.290 ± 0.026 ^**^	0.38 ± 0.05 ^*^	17.0 ± 1.8 ^*^	14.9 ± 0.5 ^**^
	556	4.01 ± 0.18 ^***^	0.93 ± 0.02 ^***^	0.371 ± 0.013 ^***^	0.51 ± 0.07 ^**^	18.2 ± 0.7 ^**^	13.5 ± 0.1 ^***^
AP	5	3.58 ± 0.18 ^***^	1.03 ± 0.05 ^**^	0.359 ± 0.020 ^***^	0.52 ± 0.01 ^***^	19.1 ± 4.0 ^**^	13.9 ± 0.3 ^***^

Data were shown as mean ± S.E.M (n = 8). ^##^ *p* < 0.01, ^###^ *p* < 0.001, compared with normal vehicle group; ^*^ *p* < 0.05, ^**^ *p* < 0.01, ^***^ *p* < 0.001, compared with hyperuricemia vehicle group.

### 2.3. Effects of FGE on Renal mRNA and Protein Levels of mOIT3 and THP Concentrations

**Figure 2:**
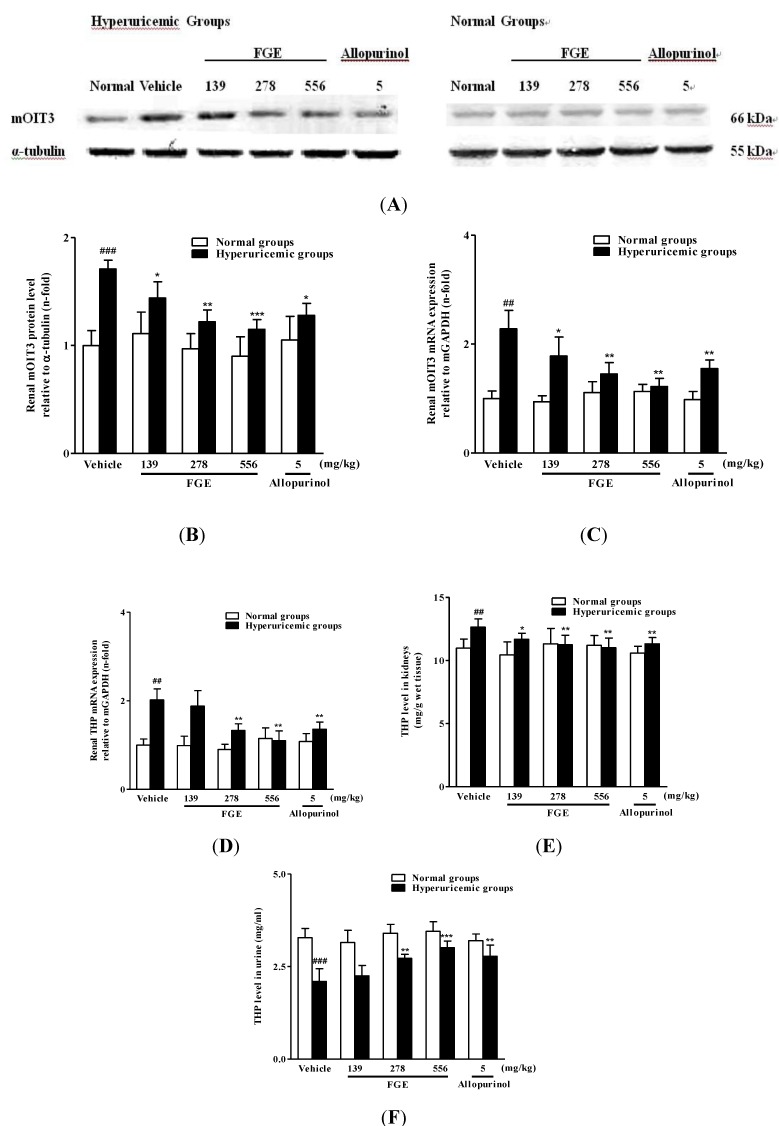
Effects of FGE and allopurinol on mRNA and protein levels of mOIT3, as well as THP levels in kidney and urine in hyperuricemic and normal mice. Kidney cortex tissues were used for western blot analysis of mOIT3 (**A**). The ratio of mOIT3 (**B**) density to α-tubulin density. Kidney cortex mRNA was extracted for RT-PCR analysis of mOIT3 (**C**) and THP (**D**). Data were expressed as the mean ± S.E.M. for four mice. THP levels in kidney (**E**) and urine (**F**) were detected by ELISA kits. Data were expressed as the mean ± S.E.M. for 8 mice. ^##^*p* < 0.01, ^###^*p* < 0.001, compared with normal vehicle animals. ^*^p < 0.05, ^**^p < 0.01, ^***^p < 0.001, compared with hyperuricemia vehicle animals.

As illustrated in Figure 2, potassium oxonate led to a remarkable up-regulation of renal mOIT3 expression compared to normal vehicle mice, which could be attenuated by FGE in a dose-dependent manner. Moreover, THP concentrations were significantly reduced in urine and increased in kidneys, while mRNA levels of renal THP were remarkably up-regulated in hyperuricemic mice. FGE at 139, 278 and 556 mg/kg effectively reversed oxonate-induced alternations of THP concentrations in mouse kidneys, while FGE at the two higher doses notably recovered renal THP mRNA levels and urinary THP concentrations in hyperuricemic mice. In addition, allopurinol at 5 mg/kg succeeded in down-regulating expressions of renal mOIT3 and modulating THP levels or expressions in kidney and urine of hyperuricemic mice. On the other hand, FGE and allopurinol had no significant effects on levels or expressions of mOIT3 and THP in normal animals.

### 2.4. Effects of FGE on Renal mRNA and Protein Levels of Organic Cation/Carnitine Transporters

**Figure 3:**
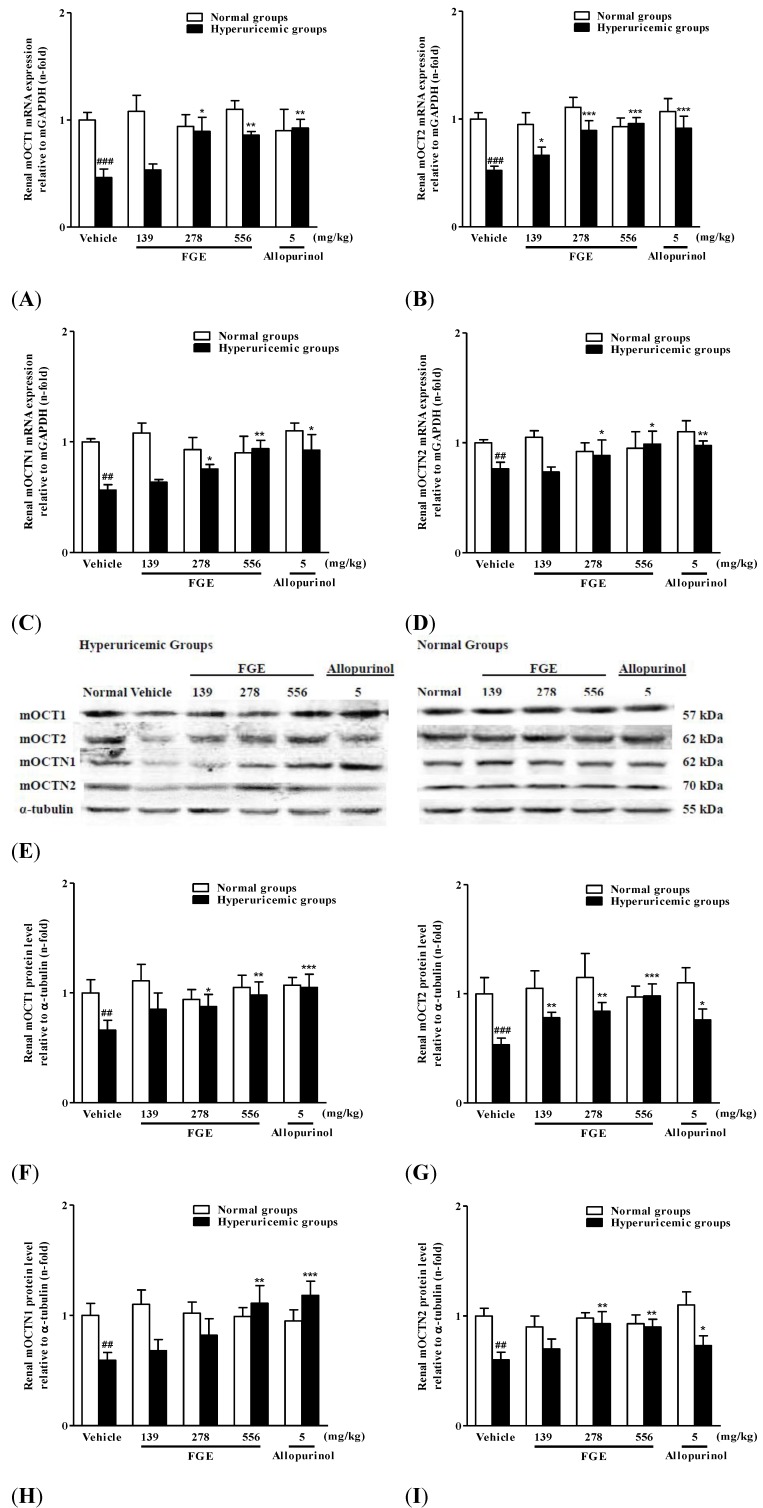
Effects of FGE and allopurinol on mRNA and protein levels of renal organic cation/cartinine transporters in hyperuricemic and normal mice. Kidney cortex mRNA was extracted for RT-PCR analysis of mOCT1 (**A**), mOCT2 (**B**), mOCTN1 (**C**) and mOCTN2 (**D**). Kidney cortex tissues were used for western blot analysis of mOCTN1, mOCTN2, mOCT1 and mOCT2 (**E**). The ratio of mOCT1 (**F**), mOCT2 (**G**), mOCTN1 (**H**) and mOCTN2 (**I**) density to α-tubulin density. Data were expressed as the mean ± S.E.M. for 4 mice. ^#^ *p* < 0.01, ^##^ *p* < 0.01, ^###^ *p* < 0.001, compared with normal vehicle animals. ^*^ *p* < 0.05, ^**^ *p* < 0.01, ^***^ *p* < 0.001, compared with hyperuricemia vehicle animals.

As shown in Figure 3, the effects of FGE and allopurinol on mRNA and protein levels of renal mOCT1, mOCT2, mOCTN1 and mOCTN2 in hyperuricemic mice. The mRNA and protein levels of renal mOCT1, mOCT2, mOCTN1 and mOCTN2 were remarkably decreased in hyperuricemic mice compared with normal vehicle group. Treatment of FGE at the two higher doses significantly up-regulated both mRNA and protein expressions of renal organic cation/carnitine transporters, except that FGE at 278 mg/kg failed to reach significance despite increased renal protein levels of mOCTN1. In addition, FGE at 139 mg/kg could also significantly increase mRNA and protein levels of renal mOCT2 in hyperuricemic mice. Moreover, allpurinol at 5 mg/kg significantly up-regulated renal expressions of mOCT1, mOCT2, mOCTN1 and mOCTN2 in this model. On the other hand, FGE or allopurinol did not significantly alter the expression levels of renal mOCT1, mOCT2, mOCTN1 and mOCTN2 in normal mice.

## 3. Discussion

FGE, currently used as diuretic in traditional Chinese medicine, has not been used clinically in gout and hyperuricemia treatment. In the present study, we firstly observed that FGE exerted hypouricemic effects by cooperatively reducing urate reabsorption through down-regulating renal mURAT1, mGLUT9 and enchancing secretion through up-regulating mOAT1 in hyperuricemic mice. Of note, expressions of mOIT3 associated with alternations of THP concentrations were firstly explored in oxonate-induced hyperuricemic mouse kidneys, which could be restored by FGE treatment. On the other hand, FGE was found to restore the expressions of renal mABCG2, mOCT1, mOCT2, mOCTN1 and mOCTN2, improving oxonate-induced renal dysfunction in mice. These findings confirmed that FGE exhibited beneficial effects on oxonate-induced hyperuricemia with renal dysfuction.

In clinic, increased serum uric acid levels induced by renal urate underexcretion contribute to about 90% of hyperuricemia and gout [5]. The enhanced protein levels of URAT1 contributed to hyperuricemia in obesity/metabolic syndrome model mice [23]. Homozygous loss-of-function mutations of URAT1 or GLUT9 result in a total defect of uric acid absorption, leading to severe renal hypouricemia [24,25]. Genetic impairment of ABCG2 increased serum uric acid levels in humans [12]. The decreased expression level of renal rOAT1 was observed in hyperuricemic rats induced by combined administration of uricase inhibitor oxonic acid and uric acid [26]. Thus, renal handling of urate transport constitutes an important target for drugs to treat hyperuricemia and gout [27]. Consistent with our previous studies, administration of potassium oxonate for seven consecutive days induced dys-expresssion of renal uric acid transporters including mURAT1, mGLUT9 and mOAT1 in the present study, subsequently leading to hyperuricemia [27,28]. It was worth noting that up-regulation of renal mABCG2 was found in oxonate-induced hyperuricemic mice in the present study, which might be explained to be an adaptive response to accumulation of urate that have to be excreted into the urine through kidney [29]. Parallel with its enhancement of renal urate excretion and reduction of serum urate in hyperuricemic mice, FGE could recover expressions of these organic anion transporters, exhibiting beneficial effects on regulating uric acid excretion. The restoration of mABCG2 in hyperuricemic mice by FGE might be contributed to its uricosuric and nephro-protective actions. Importantly, we first observed the up-regulation of mOIT3 in this model. Consistently, it was reported that OIT3 deficiency impaired uric acid reabsorption in renal tubule, indicating that OIT3 over-expression might stimulate urate reabsorption and subsequently result in hyperuricemia. Moreover, THP co-localized with OIT3 in the renal thick ascending limb of Henle’s loop was a useful marker of renal dysfunction in hyperuricemia [14], as demonstrated by some findings that mutations in THP gene lead to familial juvenile hyperuricemic nephropathy and develop kidney dysfunction in mice [30,31,32]. In the present study, obvious alternations of renal and urinary THP levels were observed in oxonate-induced hyperuricemic mice, which was in accord with dys-expression of mOIT3. Meanwhile, FGE treatment restored renal mOIT3 expressions as well as mTHP levels in kidney and urine, which might be partly due to that FGE disrupted the protein polymerization involving both mOIT3 and mTHP, promoting excretion of mTHP to the urine [14]. As those findings indicated, OIT3 provided us with some insights in exploring a new target for treating hyperuricemia. Furthermore, recent studies suggested that human OAT2 located at the basolateral membrane of proximal tubules played a role in renal uric acid uptake from blood as a first step of tubular secretion [33], while OAT4 and OAT10 in the apical membrane contributed to proximal tubular urate absorption in human kidneys [34,35]. Different from that in human, the roles of OAT2 and OAT10 in renal urate transport have not been identified while OAT4 even has no ortholog in mouse kidneys [36]. Therefore, further studies with human renal tubular epithelial cells are needed to confirm the exact mechanisms of FGE, involving OAT2, OAT4 and OAT10.

On the other hand, renal organic cation and carnitine transporters, being responsible for the uptake of endogenous substrates and detoxification of xenobiotics and chemotherapeutics, are considered to be related with renal dysfunction [37,38,39]. Many reports have touched upon the relationship between dys-expression of renal OCTs/OCTNs and renal dysfunction induced by cadmium, adenine or oxonate [17,26,27,28,40]. In the present study, FGE restored renal mRNA and protein levels of mOCT1, mOCT2, mOCTN1 and mOCTN2 in hyperuricemic mice, restoring renal function characterized by decreased serum creatinine and BUN levels, as well as increased FEUA. Therefore, we concluded that the nephro-protective effects of FGE on oxonate-treated hyperuricemic mice might be partly attributed to the regulation of renal mOCT1, mOCT2, mOCTN1 and mOCTN2 expressions. 

FGE has a variety of biological activities, but few studies have focused on its uricosuric and nephro-protective effects. Major effective constituents in FGE, such as flavonoids, geniposide, crocin and crocetin, have been demonstrated to exhibit hypouricemic effects or nephro-protective actions in animal models [27,28,41,42]. These observations suggested that, although the precise mechanisms of the uricosuric and nephro-protective effects of FGE remain incompletely understood, it might be attributed to the active principles by the regulation of renal organic ion transporters in hyperuricemia with kidney dysfunction. On the other hand, daily administration of 30g fructus gardenia resulted in hepatotoxicity, while oral administration of 1.62g/kg FGE in rats for three consecutive days led to hepatic function and morphology abnormalities [43]. In the present study, FGE treatment exhibited no side effects in normal mice at the effective doses which seemed to be much lower than the toxic dose, therefore, in consideration of the similarity of renal organic ion transporters between mice and human [11,44,45], FGE might have great potential in clinical application.

## 4. Experimental

### 4.1. Reagents

Uric acid, allopurinol and potassium oxonate were purchased from Sigma (St. Louis, MO, USA). TRIzol reagent and reverse transcriptase moloney murine leukemia virus (M-MLV) used for cDNA synthesis were obtained from Invitrogen (Carlsbad, CA, USA). SYBR green qPCR mix was purchased from Bio-rad Laboratories (Hercules, CA, USA). Assay kits of BUN, creatinine and protein concentrations were obtained from Jiancheng Biotech (Nanjing, China), respectively. ELISA assay kit of THP was purchased from R&D (Minneapolis, MN, USA).

### 4.2. Plant Material

Fructus gardenia was purchased from HuangQingRenJian drugstore (No: 1010004), and was authenticated by Prof. X.W. Lai, Jiangxi University of Traditional Chinese Medicine, Nanchang, China.

### 4.3. Preparation of the Ethanol Extract

Fructus gardeniae, 100 g, after being chopped into small pieces and ground, was dipped in 60% ethanol (800 mL), and then boiled for 2 h. The extract was immediately filtered through a two-layer mesh. The residue was again boiled in 800 mL 60% ethanol for 2 h and left to stand for 1 h before filtration. The combined filtrate was then concentrated under reduced pressure and evaporated to dryness and the yield of the extract is 35.71%.

### 4.4. Animals

All animal studies were approved by the Animal Ethics Committee of China Pharmaceutical University. Male Kun-Ming strain of mice (20 ± 2 g) purchased from the Central Institute for Experimental Animals of Zhejiang were housed 5 per cage (320 × 180 × 160 mm). Mice were maintained in a room controlled at 22–24 °C with a relative humidity of 55 ± 5% and a 12:12-h light-dark cycle (6:00 a.m.–6:00 p.m.). They were given standard chow *ad libitum* for the duration of the study and allowed 1 wk to adapt to the laboratory environment before experiments. 

### 4.5. Hyperuricemic Mice and Drug Administration

Experimental animal model of hyperuricemia induced by oral administration of potassium oxonate has been used in pharmacological studies [28,46]. Mice were divided into 10 groups: normal + vehicle, oxonate + vehicle, oxonate + 139 mg/kg FGE, oxonate + 278 mg/kg FGE, oxonate + 556 mg/kg FGE, oxonate + 5 mg/kg allopurinol (as a positive control drug), and normal + 139 mg/kg FGE, normal + 278 mg/kg FGE, normal + 556 mg/kg FGE, normal + 5 mg/kg allopurinol. Dosages of FGE and allopurinol were determined based on the adult dosages (Chinese Pharmacopoeia Committee, 2010) and our preliminary studies. According to State Pharmacopoeia of People’s Republic of China (2010 edition), dosage of fructus gardenia for adults is 6 g (the total raw materials)/day, or 780 mg raw materials/kg/day calculated by the formula that converts dosage of human into that of mouse in terms of the respective body surface areas in accordance with the Chinese Medicine Pharmacology Research Technology (1994). With a yield of 35.71%, the dose of FGE was set at 278 mg/kg/d in the present study. Therefore, we used three doses of FGE at 139, 278, 556 mg/kg in this study. 

For administration to the animals, oxonate, FGE and allopurinol were freshly dispersed in 0.9% CMC-Na solution, respectively. The solution concentrations were 16.67 mg/mL for oxonate, 9.27, 18.53, 37.07 mg/mL for FGE, and 0.33 mg/mL for allopurinol, respectively. The volume of drug administered was based on body weight measured immediately prior to each dose. Food, but not water, was withdrawn from the animals 1 h prior to the administration. Mice were administered in a volume of 15 mL/kg by gavage once daily with potassium oxonate (250 mg/kg) or 0.9% CMC-Na solution (vehicle) at 8:00 a.m. for seven consecutive days. FGE and allopurinol were orally initiated at 9: 00 a.m. on the day when the oxonate was given.

### 4.6. Blood, Urine and Tissue Sample Collection

From 24 h before final administration on the seventh day, 24 h urine sample for each mouse collected in a metabolic cage was centrifuged at 2,000 × g for 10 min to remove the particulate contaminants. Whole blood samples were collected 1 h after final administration on the seventh day and centrifuged at 10,000 × g for 5 min to obtain serum. Serum and urine samples were stored at −20 °C until biochemical assays. Simultaneously, kidney cortex tissues were rapidly and carefully removed on ice-plate, and stored at −70 °C for assays. 

### 4.7. Determination of Uric Acid and Creatinine Levels and Renal Urate Handling Parameters

Uric acid concentrations in serum (Sur) and urine (Uur) were determined by the phosphotungstic acid method [47]. Creatinine levels in serum (Scr) and urine (Ucr), as well as BUN levels were determined using trinitrophenol colorimetry or urease ultraviolet kits, respectively. FEUA was calculated using the formula: FEUA = (Uur × Scr)/(Sur × Ucr) × 100, expressed as a percentage [48].

### 4.8. Real-Time Quantitative Reverse Transcription-Polymerase Chain Reaction (RT-PCR) Analysis

Total RNA was extracted by the use of TRIzol reagent, and DNA was removed by the use of the DNA-free kit. Real-time PCR with SYBR Green involved the use of the SuperScript III Platinum Two-Step RT-PCR Kit on an ABI PRISM 7000 sequence detection PCR system (Applied Biosystems, Foster City, CA, USA). Sequences of primers were summarized in Table 2.

**Table 2:** Summary of the gene-specific RT-PCR primer sequences, the length of production and the appropriate annealing temperature used in the experiments.

Description	Genebank	primer (5′→ 3′)	Product size (bp)	Tm (°C)
mURAT1	NM_009203	GCTACCAGAATCGGCACGCT CACCGGGAAGTCCACAATCC	342	58
mGLUT9	NM_001102414	GAGATGCTCATTGTGGGACG GTGCTACTTCGTCCTCGGT	316	58
mABCG2	NM_011920	TAAATGGAGCACCTCAACCT GAGATGCCACGGATAAACTG	238	58
mOIT3	NM_010959	GCGCCATTGAAGTGAGTGTC CAGGTTGGGCACGTATCCTT	305	58
mOAT1	NM_008766	GCCTATGTGGGCACCTTGAT CTTGTTTCCCGTTGATGCGG	238	58
mOAT3	NM_031194	AAGAACATCTCTGTGAGGGTG GGCAAGATGAACCAAAACTGG	297	58
mOCT1	NM_009202	ACATCCATGTTGCTCTTTCG TTGCTCCATTATCCTTACCG	315	58
mOCT2	NM_013667	ACAGGTTTGGGCGGAAGT CACCAGAAATAGAGCAGGAAG	331	58
mOCTN1	NM_019687	AGGAGAGGTGGAAACATGCG TCCTTCGTCTCCAAGGGGAT	233	58
mOCTN2	NM_011396	CTTATTCCCATACGGGCGCT TTTCTGAGGCACCTGTCGTC	279	58
mGAPDH	NM_008084	TGAGGCCGGTGCTGAGTATGT CAGTCTTCTGGGTGGCAGTGAT	299	58

### 4.9. Western Blot Analysis of mURAT1, mABCG2, mGLUT9, mOIT3, mOAT1, mOCT1, mOCT2, mOCTN1, mOCTN2

Mouse kidney cortex for mURAT1, mABCG2, mGLUT9, mOAT1, mOAT3, mOIT3, mOCT1, mOCT2 and α-tubulin analysis was homogenized in 1 mL RIPA buffer using a Polytron set, and centrifuged at 3000 × *g* for 15 min (4 °C). The supernatant was centrifuged at 12,000 × g for 20 min, 4 °C to get tissue supernatant. Protein concentrations of kidney cortex supernatant were determined by the Bradford protein assay reagent (Jiancheng Biotech, Nanjing, China) with bovine serum albumin as standard. western Blot analyses for renal mURAT1, mABCG2, mGLUT9, mOIT3, mOAT1, mOAT3, mOCT1, mOCT2, mOCTN1, mOCTN2 and α-tubulin were described in our previous studies [27,42]. Primary antibodies were listed in Table 3.

**Table 3:** Antibodies used for western blot analyses.

Company	Description	Catalog number
SaiChi Biotech	Rabbit murat1 antibody	001052-R
(Beijing, China)	Rabbit mglut9 antibody	001046-R
	Rabbit moat1 antibody	001019-R
	Rabbit moat3 antibody	001020-R
	Rabbit moct1 antibody	001017-R
	Rabbit moct2 antibody	001018-R
Alpha Diagnostic International	Rabbit moctn1 antibody	OCTN11-A
Inc. (San Antonio, TX, USA)	Rabbit moctn2 antibody	OCTN21-A
Cell Signaling Technology Inc.		
(Boston, MA, USA)	Rabbit α-tubulin antibody	#2148
Epitomics Inc.	Rabbit moit3 antibody	6862-1
(Hangzhou, China)	Goat anti-rabbit igg HRP	3056-1

### 4.10. Determination of THP Levels

Mouse kidney tissues were homogenized in 10 wt/vol of sodium chloride on ice and then centrifuged (10,000 × *g*) for 15 min at 4 °C. THP levels in serum, urine and kidney were analyzed by ELISA kits, respectively. Fifty µL standard, control or sample mixed with 50 µL assay diluent were added to each well and incubated at room temperature for 2 h. After removing unbound material by washing five times, 100 µL of conjugate was added to each well and incubated at room temperature for 2 h. After rinsing each well, 100 µL substrate solution was added and incubated at room temperature away from light. 30 min later, the reaction was stopped by adding 100 µL of stop solution to each well, and optical density was read at 450 nm within 30 min in enzyme-labeled instrument.

### 4.11. Statistical Analysis

All data were expressed as means ± SD. Statistical differences between means were determined using analysis of variance on ranks followed by a post hoc test (LSD procedures) as appropriate. A value of *p* < 0.05 was considered statistically significant. Figures were obtained by the Statistical Analysis System (GraphPad Prism 5, GraphPad Software, Inc., San Diego, CA, USA).

## 5. Conclusion

In conclusion, the present study has demonstrated for the first time that FGE exhibited potently uricosuric effects in hyperuricemic mice by down-regulating renal mURAT1, mGLUT9 expressions and up-regulating renal mOAT1 expression. Moreover, we firstly found that renal mOIT3 dys-expressions participated in the pathogenesis of oxonate-induced hyperuricemia, which could be reversed by FGE treatment. On the other hand, FGE improved renal dysfunction in hyperuricemia by recovering renal expression of mABCG2, mOCT1, mOCT2, mOCTN1 and mOCTN2 in mice. The molecular mechanisms by which FGE displays the uricosuric and nephro-protective actions in oxonate-treated mice provide some scientific support for the empirical use of Fructus Gardenia as a traditional Chinese medicine for hyperuricemia with renal dysfunction.
